# Supplementary material for: Molecular Dynamics of Lysine Dendrigrafts in Methanol–Water Mixtures
Source: Int J Mol Sci. 2023 Feb 4;24(4):3063. doi: 10.3390/ijms24043063 (PMC9963150; doi:10.3390/ijms24043063)
Supplement: Supplementary file 1 [file ijms-24-03063-s001.zip › ijms-2165228-supplementary.pdf]

International Journal of
Molecular Sciences

MDPI

Article

Molecular dynamics of lysine dendrigrafts in methanol-water mixtures. Supporting Information.

Emil I. Fatullaev [1], Oleg V. Shavykin [1,2,3] and Igor M. Neelov [1,]*

[1] School of Computer Technologies and Control, St. Petersburg National Research University of Information Technologies, Mechanics and Optics (ITMO University), Kronverkskiy pr. 49, St. Petersburg, 197101, Russia
[2] Physics Department, Lomonosov Moscow State University, Leninskie Gory 1-2, Moscow, 119991, Russia
[3] Department of Mathematics, Tver State University, Sadoviy per., 35, 170102 Tver, Russia
* Correspondence: i.neelov@mail.ru

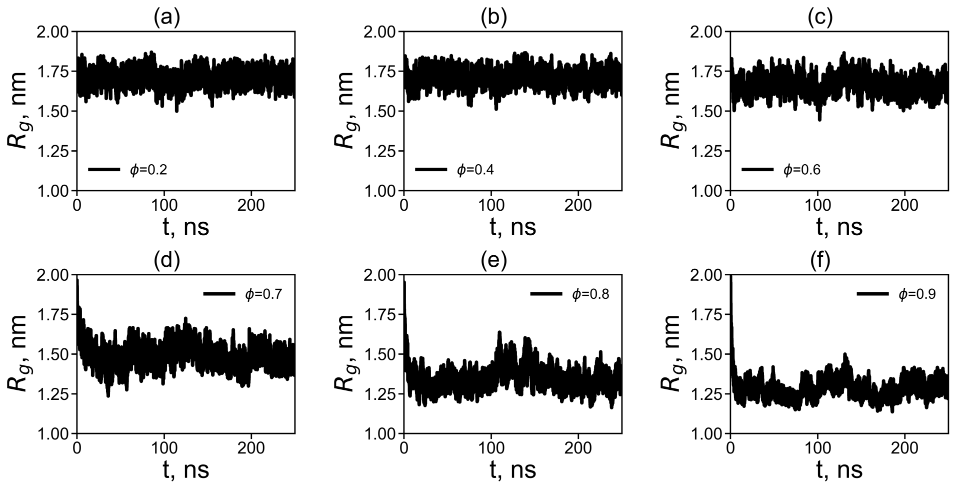

Figure S1. The mean-squared radius of gyration R_g as function of time for the equilibration run of DGL2 at different methanol fraction ϕ. Upper pictures from left to right: 0.2, 0.4 and 0.6; bottom pictures from left to right: $\phi = 0.7, 0.8, 0.9$.

Citation: Fatullaev, E.I.; Shavykin, O.V.; Neelov, I.M. Molecular dynamics of lysine dendrigrafts in methanol-water mixtures. Supporting Information. *Int. J. Mol. Sci.* **2023**, *24*, 3063. https://doi.org/10.3390/ijms24043063

Received: 30 December 2022
Revised: 22 January 2023
Accepted: 27 January 2023
Published: 4 February 2023

Copyright: © 2023 by the authors. Submitted to *Int. J. Mol. Sci.* for possible open access publication under the terms and conditions of the Creative Commons Attribution (CC BY) license (https://creativecommons.org/licenses/by/4.0/).
